# An assessment of ventilator-associated pneumonias and risk factors identified in the Intensive Care Unit

**DOI:** 10.12669/pjms.324.10381

**Published:** 2016

**Authors:** Mevlut Karatas, Sedat Saylan, Ugur Kostakoglu, Gurdal Yilmaz

**Affiliations:** 1 Mevlut Karatas, Assistant Professor, Department of Pulmonology, Faculty of Medicine, Recep Tayyip Erdogan University, Rize, Turkey; 2 Sedat Saylan, Assistant Professor, Department of Anesthesiology and Reanimation, Faculty of Medicine, Karadeniz Technical University, Trabzon, Turkey; 3 Ugur Kostakoglu, Assistant Professor, Department of Infection Diseases and Clinic Microbiology, Faculty of Medicine, Recep Tayyip Erdogan University, Rize, Turkey; 4 Gurdal Yilmaz, Associate Professor, Department of Infection Diseases and Clinic Microbiology, Faculty of Medicine, Karadeniz Technical University, Trabzon, Turkey

**Keywords:** Ventilator associated pneumonia, Intensive care unit, Infection

## Abstract

**Objectives:**

Ventilator-associated pneumonia (VAP) is a significant cause of hospital-related infections, one that must be prevented due to its high morbidity and mortality. The purpose of this study was to evaluate the incidence and risk factors in patients developing VAP in our intensive care units (ICUs).

**Methods:**

This retrospective cohort study involved in mechanically ventilated patients hospitalized for more than 48 hours. VAP diagnosed patients were divided into two groups, those developing pneumonia (VAP(+)) and those not (VAP(-)).\

**Results:**

We researched 1560 patients in adult ICUs, 1152 (73.8%) of whom were mechanically ventilated. The MV use rate was 52%. VAP developed in 15.4% of patients. The VAP rate was calculated as 15.7/1000 ventilator days. Mean length of stay in the ICU for VAP(+) and VAP(-) patients were (26.7±16.3 and 18.1±12.7 days (p<0.001)) and mean length of MV use was (23.5±10.3 and 12.6±7.4 days (p<0.001)). High APACHE II and Charlson co-morbidity index scores, extended length of hospitalization and MV time, previous history of hospitalization and antibiotherapy, reintubation, enteral nutrition, chronic obstructive pulmonary disease, cerebrovascular disease, diabetes mellitus and organ failure were determined as significant risk factors for VAP. The mortality rate in the VAP(+) was 65.2%, with 23.6% being attributed to VAP.

**Conclusion:**

VAPs are prominent nosocomial infections that can cause considerable morbidity and mortality in ICUs. Patient care procedures for the early diagnosis of patients with a high risk of VAP and for the reduction of risk factors must be implemented by providing training concerning risk factors related to VAP for ICU personnel, and preventable risk factors must be reduced to a minimum.

## INTRODUCTION

Intensive care units (ICUs) are life support units intended to care for patients requiring intensive care due to organ failure, that are equipped with advanced technology, where vital signs are monitored and where treatment is administered.^1^ The majority of patients monitored in these units receive mechanical ventilation (MV) support and invasive procedures such as central venous catheterization. However, patients develop a disposition to infections as a result of these procedures.^2^ Ventilator-associated pneumonia (VAP) is the most common infection in intensive care patients, and can lead to prolongation of intensive care and an increased risk of mortality.^2^ Compromise of patient defense mechanisms, colonization by pathogen micro-organisms and the presence of micro-organisms with high virulence all occupy and important place in the pathogenesis of VAP.

The purpose of this study was to determine the incidence and risk factors in patients developing VAP in our ICUs.

## METHODS

We received permission for present study from local ethics committee of Kanuni Education and Research Hospital, Turkey. and the study was performed retrospectively at the same hospital, which has a 605-bed capacity, including 46 adult ICU beds. Patients hospitalized in the ICU for longer than 48 hour and administered MV between January 1, 2011 and 31st December 2013 were included in the study. Our hospital contains four adult ICUs (Anesthesia and Reanimation, Surgical, Medical and Neurology). Due to nurse shortages, the nurse-patient ratio in our ICUs ranges between 1:3 and 1:4, and may even rise to 1:6 on some nights. Patients’ demographic and clinical characteristics were recorded onto study forms by examination of medical files, infection control committee surveillance data, ICU records, pharmacy records and processing data.

The Acute Physiology and Chronic Health Evaluation (APACHE) II scores used were those calculated in the first 24 hour of hospitalization.^3^ Charlson co-morbidity index scores were obtained by examining all patients’ medical records.^4^ Identification of microorganisms and testing for antimicrobial susceptibility were conducted using the Phoenix system (Becton Dickinson), the disk diffusion test, and classic methods. Patients’ demographic and clinical characteristics (APACHE II score, Charlson co-morbidity index. Length of hospitalization, treatments administered and invasive procedures performed) and prognoses were recorded. VAP was diagnosed on the basis of CDC criteria.^5^ Patients were divided into two groups, those developing pneumonia (VAP(+)) and those not developing pneumonia (VAP(-)).

### Statistical Analysis

Descriptive statistical analysis was performed for all parameters. The Kolmogorov-Smirnov test was used to determine the eligibility of variables. Data in conformity with normal distribution were analyzed using Student’s t-test, and those not conforming to normal distribution were analyzed using the Mann Whitney-U test. Data obtained by measurements are given as mean ± standard deviation. Data obtained by counting are given as numbers (%); analyses were performed using the Chi-square test. P<0.05 was regarded as significant.

## RESULTS

MV was administered to 1152 (73.8%) of the 1560 patients with an ICU stay exceeding 48 hour. The MV use rate was 0.52. Two hundred fourteen VAP attacks occurred in 178 patients (15.4%) receiving MV. The VAP rate was 15.7 in 1000 ventilator days, and mean time to development of VAP was 13.2±8.6 days. Mean age of the VAP(+) patients was 67.8±21.1 and mean age of the VAP(-) patients was 69.4±18.1 (p=0.864). Mean length of stay in the ICU in VAP(+) patients was 26.7±16.3 days, and mean length of MV was 23.5±10.8 days. Mean length of stay in the ICU in VAP(-) patients was 18.1±12.7 days, and mean length of MV was 12.6±7.4 days (p<0.001) (Table-I).

**Table-I:** Assessment of risk factors for development of VAP.

Variables	VAP(+) n=178 (%)	VAP(–) n=974 (%)	P value	OR	95% CL
Age	67.8±21.1	69.4±18.1	0.864		
Gender(Male)	102(57.3)	526(54.0)	0.416	1.14	0.82-1.60
APACHE II	21.5±5.4	19.2±4.9	<0.001		
Charlson co-morbidity index	3.9±1.6	2.7±3.0	<0.001		
Length of hospitalization (days)	26.7±16.3	18.1±12.7	<0.001		
Length of ventilation (days)	23.5±10.8	12.6±7.4	<0.001		
Previous history of hospitalization	63 (35.4)	191(19.6)	<0.001	2.25	1.57-3.22
Previous history of antibiotherapy	81 (45.5)	287(29.5)	<0.001	2.00	1.42-2.80
Steroid treatment	46 (25.8)	235(24.1)	0.624	1.10	0.75-1.60
Surgical procedure	44 (24.7)	286(29.4)	0.208	0.79	0.54-1.16
Reintubation	49 (27.5)	38 (3.9)	<0.001	9.36	5.75-15.24
Enteral nutrition	146 (82.0)	611(62.7)	<0.001	2.71	1.78-4.15
**Underlying Diseases:**					
Trauma	57 (32.0)	254(26.1)	0.100	1.34	0.93-1.91
COPD	40 (22.5)	63 (6.5)	<0.001	4.19	2.65-6.62
Cardiac disease	11 (9.6)	49 (5.0)	0.652	1.24	0.60-2.53
Cerebrovascular disease	72 (40.4)	295(30.3)	0.007	1.56	1.11-2.20
Diabetes mellitus	35 (19.7)	113(11.6)	0.003	1.86	1.20-2.89
Renal disease	27 (15.2)	126(12.9)	0.492	1.20	0.75-1.93
Organ failure	38 (18.5)	132(13.6)	0.007	1.73	1.13-2.64
Malignancy	21 (11.8)	98 (10.1)	0.571	1.20	0.70-2.02
Infectious disease	57 (32.0)	244(25.1)	0.052	1.41	0.98-2.02
Mortality	116 (65.2)	512(52.6)	0.002	1.69	1.19-2.39

Mean APACHE II score in the VAP(+) patients was 21.5±5.4 and mean APACHE II score in the VAP(-) patients was 19.2±4.9. APACHE II score elevation was statistically significantly correlated with VAP development (p<0.001). Charlson co-morbidity index in the VAP(+) patients was 3.9±1.6, compared to 2.7±3.0 in the VAP(-) patients. A statistically significant correlation was observed between Charlson co-morbidity index elevation and VAP development (p<0.001)

In terms of underlying diseases, chronic obstructive pulmonary disease (COPD), diabetes mellitus (DM), cerebrovascular disease and organ failure levels differed significantly between the two groups (p<0.001, p=0.003, p=0.007, p=0.007). Previous hospitalization, a history of antibiotherapy, reintubation and enteral nutrition were assessed as significant risk factors for VAP (p<0.001).

Gram negative bacteria were isolated at a level of 78.9% from endotracheal material from patients with VAP, Gram positive bacteria at 19.4% and fungi at 1.7%. Polymicrobial growth was determined in 4.2% of VAPs. The five most common causes of VAP were *Acinetobacter species* (31.0%), *Pseudomonas aeruginosa* (27.6%), *Staphylococcus aureus* (15.1%), *Klebsiella species* (6.5%) and *Escherichia coli* (5.6%) (Table-II). Bacteremia was determined concurrently with VAP in 15.7% of patients.

**Table-II:** Identified agents in the etiology of ventilator-associated pneumonia.

Microorganisms	n (%)
Acinetobacter baumannii	72 (31.0)
Pseudomonas aeruginosa	64 (27.6)
Staphylococcus aureus	35 (15.1)
Klebsiella spp	15 (6.5)
Escherichia coli	13 (5.6)
Enterobacter spp	9 (3.9)
Enterococcus spp	6 (2.5)
Stenotrophomonas maltophilia	5 (2.2)
Serratia marcescens	5 (2.2)
Streptococcus pneumoniae	4 (1.7)
Candida albicans	4 (1.7)

One hundred sixteen (65.2%) of the patients diagnosed with VAP died. Mortality attributed to VAP was 23.6% (n=42). In addition, 52.6% of VAP(-) patients died. A statistically significant correlation was observed between mortality of VAP(+) and VAP(-) patients (p=0.002).

## DISCUSSION

MV in ICUs is a life-saving medical procedure in the event of respiratory failure. More than 300,000 patients in the USA receive MV every year.^1^ According to an American Thoracic Society (ATS) report, the prevalence of VAP ranges between 9% and 27%.^2^ A study from France reported a level of 14.5-27.6%.^6^ In our study, VAP developed in 15.4% of patients administered MV, which is compatible with the literature.

Centers for Disease Control and Prevention data report an incidence of VAP of 0.0-5.8/1000 ventilator days in the ICUs of various hospitals.^5^ However, the incidence of VAP reported in studies in the literature is as high as 58.^7^,^8^ The incidence of VAP in our study was 15.7/1000 ventilator days. Although our findings are higher than that CDC data, they are better than those of other studies. The presence of various negative factors in terms of infection, such as the fact that our hospital data were obtained from ICUs in four different branches, the high number of patients per nurse in the ICU, the lack of isolation rooms, the low square meter area per bed and the distance between beds being less than two meters may be reasons for the incidence of VAP differing from the CDC.

VAP prolongs length of hospitalization and duration of MV^2^. Mean duration of MV and length of stay in the ICU in this study were higher in patients with VAP than in VAP(-) patients (p<0.001). Every day that patients spend in the ICU and on MV increases the risk of infection. Factors facilitating infection include underlying diseases, comorbid factors, malnutrition, nasogastric tube use, gastroesophageal reflux, sedation, invasive procedures to the respiratory system and aspiration of contaminated secretions accumulating on the endotracheal cuff.^9^,^10^ MV indications in patients hospitalized in the ICU must therefore be assessed daily, and patients must be removed from MV and the ICU as quickly as possible.

APACHE II scoring is a system used to measure the severity of diseases in ICUs.^3^ Though many studies have identified severity of underlying diseases as a potential risk factor, contradictory results have also been reported. While some studies have reported that APACHE II score is associated with mortality but not with infection, other studies have suggested that a high APACHE II score is a risk factor for VAP.^3^,^11^ Apostolopoulou reported that a score of 18 or higher is an independent risk factor for VAP, and Meric et al. reported that APACHE II score is not a risk factor for hospital-acquired infection but that it is a risk factor for mortality.^12^,^13^ In our study, high APACHE II score emerged as a risk factor for VAP (p<0.001). Charlson co-morbidity index, the total score of co-morbid diseases, was 3.9±1.6 for the VAP(+) patients and 2.7±3.0 for the VAP(-) patients. There was also a statistical significance between a high Charlson co-morbidity index and VAP (p<0.001). This indicates that underlying diseases and the presence of severe disease increase the risk of VAP.

Prolongation of stay in intensive care patients and a history of recurrent hospitalization are reported to affect development of infection.^14^,^15^ Meric et al. reported that hospitalization longer than seven days increases the risk of infection.^13^ A case-control study by Agarwal reported that a mean hospitalization time of 13 days for VAP(+) patients and 8 days for VAP(-) subjects.^14^ In our study, prolonged stay in the ICU and a history of recurrent hospitalization increased the risk of VAP (p<0.001, OR=2.25).

Patients monitored in the ICU receive antibiotics for postoperative surveillance, for prophylactic reasons based on infections, and for pre-emptive as well as therapeutic purposes. Off-label and inappropriate length of use of prophylactic antibiotics are not recommended since this will increase colonization by resistant pathogens and the risk of infection.^12^ Some studies have shown that antibiotic use increases the risk of VAP risk, although other studies have reported conflicting results results.^10^,^15^ In our study, a previous history of antibiotics increased therisk of VAP 2-fold (p<0.001, OR=2.0).

Recurrent intubations increase the risk of VAP by leading to the aspiration of nosocomial bacteria colonizing the oropharynx.^16^ Therefore, instead of reintubation of an extubated patient, non-invasive MV should be applied as far as possible.^17^ Karthikeyan et al. has reported that reintubation was an important risk factor for VAP.^7^ In our study, reintubation increased the risk of VAP 9.36-fold (p<0.001, OR=9.36).

Although enteral nutrition is recommended for intensive care patients, it has been reported as a risk factor for VAP in several studies.^18^,^19^ This may be related to issues such as enteral nutrition technique, ineffective follow-up of gastric residual volume, frequent nasogastric procedures, an inappropriate patient head position during nutrition, and inadequate tracheal cuff pressure. In our study, enteral nutrition increased the risk of VAP 2.71-fold (p<0.001, OR=2.71).

Considering VAP development in terms of primary and underlying diseases, we observed significantly more VAP development in patients with disease, such as COPD, DM and organ failure(p=0.007 for organ failure, p=0.003 for DM). Previous studies have reported that underlying diseases, and particularly COPD and ARDS, lead to gram negative bacteria colonization, affect the mucociliary system, impair local and systemic defense mechanisms and affect the phagocytic functions of alveolar macrophages as well as neutrophils, thus leading to an increase in VAP development.^20^ Some studies have reported a correlation between COPD and VAP, although other studies do not describe COPD as a risk factor.^14^,^18^,^21^ In our study, COPD increased the risk of VAP 4.19-fold times (p<0.001, OR=4.19).

Organ failures may predispose for VAP in association with deterioration of underlying condition and facilitation of bacterial translocation.^22^ In addition to studies reporting no relation between organ failure and devlopment of VAP, Agarwal et al. reported a relation between chronic kidney disease and VAP.^14^,^18^ In our study, three diseases (heart failure, renal failure, and hepatic failure) were identified as a risk factor for VAP, increasing VAP development 1.73-fold (p=0.007, OR=1.73). DM was also a risk factor for VAP and increased VAP development 1.86-fold (p=0.003, OR=1.86). Arozullah et al.^23^ identified DM as a risk factor, but Agarwal et al study did not.^14^

VAP has a direct effect on mortality in hospital-associated infections. Bacteremia (particularly Pseudomonas aeruginosa or Acinetobacter spp.), medical diseases, severity of primary disease, inadequate empirical treatment, prolonged hospitalization, and advanced age are reported to increase mortality rate.^2^,^6^,^10^,^22^ In their meta-analysis, Melsen et al. reported a level of mortality attributable to VAP in surgical patients of 69%, and a level of mortality attributable to VAP of 36% in patients with intermediate severity of illness scores.^24^ In our study, 65.2% of patients with VAP died, and the level of mortality attributable to VAP was 23.6%.

The majority of VAP agents are microorganisms with high antibiotic resistance, such as Pseudomonas aeruginosa, Acinetobacter baumannii and Staphylococcus aureus.^14^,^15^,^17^ One prospective study from Italy isolated Acinetobacter baumannii (61.9%), *Pseudomonas aeruginosa* (22.5%), *Enterococcus fecalis* (4.2%) and *Candida albicans* (4.2%) as VAP agents.^25^ The five most common causes of VAP in our study were *Acinetobacter species* (31.0%), *Pseudomonas aeruginosa* (27.6%), *Staphylococcus aureus* (15.1%), *Klebsiella species* (6.5%) and *Escherichia coli* (5.6%). These factors, known as multiple resistant pathogens, are generally involved in late onset of VAP. Ninety five percent of VAP in this study was late onset.

## CONCLUSION

VAPs are nosocomial infections that cause significant morbidity and mortality in ICUs and that prolong hospitalization. These infections are more common in patients with APACHE II score and Charlson co-morbidity index elevation, with extended hospitalization and MV use and with underlying predisposing diseases. Reintubation increases the risk of VAP 9.3-fold. Guidelines must be adopted in the prevention of these infections, and every country, hospital and ICU must adopt infection control procedures in the light of its own local problems. Training must be provided for ICU personnel on the subject of VAP-related risk factors. Patients’ MV requirements must be assessed daily. The probability of reintubation must be reduced to a minimum, and prolonged MV must be prevented. Patients at high risk for VAP must be diagnosed early and patient care procedures to reduce risk factors must be implemented, and preventable risk factors must be reduced to a minimum.
